# Accuracy of telephone triage for predicting adverse outcomes in suspected COVID-19: an observational cohort study

**DOI:** 10.1136/bmjqs-2021-014382

**Published:** 2022-03-30

**Authors:** Carl Marincowitz, Tony Stone, Peter Bath, Richard Campbell, Janette Kay Turner, Madina Hasan, Richard Pilbery, Benjamin David Thomas, Laura Sutton, Fiona Bell, Katie Biggs, Frank Hopfgartner, Suvodeep Mazumdar, Jennifer Petrie, Steve Goodacre

**Affiliations:** 1 Centre for Urgent and Emergency Care Research (CURE), Health Services Research School of Health and Related Research (ScHARR), University of Sheffield, Sheffield, UK; 2 Centre for Health Information Management Research (CHIMR) and Health Informatics Research Group, Information School, University of Sheffield, Sheffield, UK; 3 Yorkshire Ambulance Service NHS Trust, Wakefield, UK; 4 Clinical Trials Research Unit (CTRU), Health Services Research School of Health and Related Research (ScHARR), The University of Sheffield, Sheffield, UK

**Keywords:** COVID-19, risk management, ambulatory care, prehospital care

## Abstract

**Objective:**

To assess accuracy of telephone triage in identifying need for emergency care among those with suspected COVID-19 infection and identify factors which affect triage accuracy.

**Design:**

Observational cohort study.

**Setting:**

Community telephone triage provided in the UK by Yorkshire Ambulance Service NHS Trust (YAS).

**Participants:**

40 261 adults who contacted National Health Service (NHS) 111 telephone triage services provided by YAS between 18 March 2020 and 29 June 2020 with symptoms indicating COVID-19 infection were linked to Office for National Statistics death registrations and healthcare data collected by NHS Digital.

**Outcome:**

Accuracy of triage disposition was assessed in terms of death or need for organ support up to 30 days from first contact.

**Results:**

Callers had a 3% (1200/40 261) risk of serious adverse outcomes (death or organ support). Telephone triage recommended self-care or non-urgent assessment for 60% (24 335/40 261), with a 1.3% (310/24 335) risk of adverse outcomes. Telephone triage had 74.2% sensitivity (95% CI: 71.6 to 76.6%) and 61.5% specificity (95% CI: 61% to 62%) for the primary outcome. Multivariable analysis suggested respiratory comorbidities may be overappreciated, and diabetes underappreciated as predictors of deterioration. Repeat contact with triage service appears to be an important under-recognised predictor of deterioration with 2 contacts (OR 1.77, 95% CI: 1.14 to 2.75) and 3 or more contacts (OR 4.02, 95% CI: 1.68 to 9.65) associated with false negative triage.

**Conclusion:**

Patients advised to self-care or receive non-urgent clinical assessment had a small but non-negligible risk of serious clinical deterioration. Repeat contact with telephone services needs recognition as an important predictor of subsequent adverse outcomes.

**Key messages**

**What is already known on this topic**

- Telephone triage has been used to divert patients with suspected COVID-19 to self-care or for non-urgent clinical assessments, and thereby help mitigate the risk of health services being overwhelmed by patients who require no specific treatment.
- Concerns have been raised that telephone triage may not be sufficiently accurate in identifying need for emergency care; however, no previous evaluation of accuracy of telephone triage in patients with suspected COVID-19 infection has been completed.

**What this study adds**

- Patients advised to self-care or receive non-urgent clinical assessment had a small but non-negligible risk of deterioration and significant adverse outcomes.
- Telephone triage has comparable performance to methods used to triage patient acuity in other emergency and urgent care settings.
- Accuracy of triage may be improved by better recognition of multiple contact with services as a predictor of adverse outcomes.

**Key messages**

How this study might affect research, practice or policy

- Telephone triage can have an important role in managing lower-risk patients during the COVID-19, and potentially future, pandemics and help prevent patients who require no specific treatment from attending hospitals or other care providers.
- The under-recognised risk of deterioration associated with multiple contacts with telephone triage services has been fed back to the service provider to be incorporated in risk stratification.

## Background

During the COVID-19 pandemic, there was a risk that hospitals could be overwhelmed by patients who did not need specific treatment. UK government pandemic planning predicted that, in the advent of an influenza or similar pandemic, there could be around 750 000 excess emergency department (ED) attendances in the UK.^1 2^ Attendances were predicted to be largely for patients who would not require hospitalisation.^3 4^

To reduce this risk, from 18 February 2020 onwards, NHS England advised patients with suspected infection to contact the National Health Service (NHS) 111 service instead of attending healthcare providers.^5^ NHS 111 is a national, free-to-use 24-hour telephone triage service for urgent health problems. Initial triage is carried out by trained, non-clinical call advisors using the NHS Pathways clinical decision support software. The end point (disposition) is advice on what to do next, in terms of which service to access and the timeframe within which this access should occur. If appropriate, the call can be passed onto a clinician (usually a nurse or paramedic) for further assessment and, depending on local arrangements, callers can speak to other specialist clinicians or appointments can be made with relevant services, including general practitioners. Similar COVID-19 telephone triage ‘hotlines’ have been implemented in parts of the USA.^6 7^

In the first 6 months of the COVID-19 pandemic, ED attendances in the UK decreased by approximately 25%, probably due, at least in part, to displacement of care.^8^ Patients who did attend the ED with suspected COVID-19 infection were high acuity with a mortality rate of 15.5%, with lower acuity patients likely being managed via NHS 111.^9^ Indeed, there were almost 3 million NHS 111 calls made across England in March 2020; a record number and double the number in March for the previous year.^10^ To cope with the increase in call volume, a specific telephone triage pathway for patients with suspected COVID-19 infection was introduced in early February 2020, which underwent rapid updates as the pandemic progressed. Local NHS 111 services used interim triage methods while awaiting implementation of new telephone triage pathways and, due to excess demand, calls started to be diverted to a national centre on 4 March 2020.

Concerns have been raised that during this period of high demand and reconfiguration of services, telephone triage may have underappreciated the severity of some callers’ illness, leading to delays in treatment and avoidable harm.^11^ There have been calls for an inquiry into the effectiveness of NHS 111 telephone triage at identifying critically unwell patients and the Healthcare Safety Investigation Branch (HSIB) has started an investigation into NHS 111’s response to callers with suspected COVID-19.^12 13^ A specific concern raised by public and patient representatives affiliated with HSIB is: ‘The NHS 111 telephone advice given did not fully respond to the severity of the reported symptoms’.^13^

There has been no previous evaluation of the accuracy of the clinical risk-assessment performed by this service nor, to our knowledge, other telephone triage services for patients with suspected COVID-19 infection. Evaluating the accuracy of telephone triage and specifically estimating the risk of serious adverse outcome in those advised to self-care or wait for non-urgent assessment allows safety concerns regarding underappreciation of illness severity to be examined.

Our study aimed to:

1. assess how accurately NHS 111 telephone services identified those who suffered an adverse outcome needing an emergency response;
2. identify any factors that may have affected the accuracy of telephone triage.

## Methods

### Study design

The Pandemic Respiratory Infection Emergency System Triage (PRIEST) study was piloted as the Pandemic Influenza Triage in the Emergency Department (PAINTED) study, part of the National Institute for Health Research portfolio of studies to be activated in an influenza pandemic in England.^14^ However, it was adapted in February 2020 in response to the COVID-19 pandemic, to include an expanded range of respiratory infections and evaluate prehospital urgent and emergency care triage services. This evaluation of NHS 111 telephone services is an observational cohort study that forms part of the PRIEST study and is reported in accordance with the REporting of studies Conducted using Observational Routinely-collected health Data Statement guidance.^15^

### Setting

Yorkshire Ambulance Service NHS Trust (YAS) provides 24-hour emergency and healthcare services for the Yorkshire and Humber, Bassetlaw, North Lincolnshire and Northeast Lincolnshire region in the north of England; an area of approximately 6000 square miles and with a population of 5.3 million. In 2018/19, YAS received >998 500 emergency medical service dispatch and 1 632 514 NHS 111 calls.

### Data sources and data linkage

YAS provided a dataset of NHS 111 calls, triaged using an assessment pathway indicating possible COVID-19 infection, received between 18 March 2020 and 29 June 2020. This timeframe was selected to encompass the ‘first wave’ of the COVID-19 pandemic in England (March to June 2020) and due to the extension of NHS 111 online triage services for suspected COVID-19 in June 2020, including scheduling of clinical assessments.^16 17^ All patients within the English NHS are allocated a unique identification number, the NHS number. Records with no NHS number (<2%) were not provided as these records could not be associated with a traceable individual without manual review. The dataset consisted of patient identifiers, demographic data, call details and triage dispositions extracted from routinely collected electronic NHS 111 call records (online supplemental material 1).

10.1136/bmjqs-2021-014382.supp1 Supplementary data

Patient identifiers were provided to NHS Digital for them to trace the identities of our cohort (ie, indicate different sets of identifiers belonging to the same patient) and to supply additional individual-level demographic, comorbidity and outcome data. NHS Digital manages national health and care data collections from a variety of settings and providers in England.^18^ NHS Digital identified records in their collections belonging to patients in our cohort and provided data on patient demographics, limited COVID-related general practice (GP) records, ED attendances, hospital inpatient admissions, critical care periods and death registrations from the Office for National Statistics (online supplemental material 2).

Both YAS and NHS Digital removed records belonging to patients who had registered an NHS national data opt-out. The study team excluded patients who had opted out of any part of the PRIEST study and those with inconsistent records (eg, multiple deaths recorded or death before latest activity). Patient identifiers across all datasets were replaced with a consistent pseudo-identifier to enable the identification of records belonging to individual patients across datasets without revealing patient identifiers.

### Inclusion criteria

Our final cohort consisted of all adult (aged 16+ years) patients at time of first call (index contact) within the YAS NHS 111 calls dataset who were traced by NHS Digital and for whom a final triage disposition, and therefore urgency of recommended triage, was recorded for their index contact.

### Patient characteristics

Comorbidities recorded 12 months before the index contact with NHS 111 were extracted from electronic healthcare data provided by NHS digital (online supplemental material 2). This is consistent with the timescale for inclusion of comorbidities used to calculate comorbidity indexes using other routine data sources.^19 20^ Immunosuppressant drug use only contributes to the immunosuppression comorbidity if recorded in the 30 days before index contact. Pregnancy status was based on GP records recorded in the previous 9 months. Frailty in patients older than 65 years was derived from the latest recorded (if any) clinical frailty scale score present in the electronic GP records prior to index contact.^21^ Smoking status was similarly derived from GP records based on the latest recorded (if any) smoking status prior to the index contact.

### Outcomes

The primary outcome was death or renal, respiratory or cardiovascular organ support (serious adverse outcomes) at 30 days from index contact (identified from death registrations and critical care data).

The secondary outcome was death or organ support at 3 and 7 days from index contact.

### Analysis

We first conducted a descriptive analysis of patient demographics, comorbidities and call disposition and used multivariable logistic regression modelling to confirm known patient characteristics associated with the primary adverse outcome in COVID-19 infection. The model included: age, gender, available comorbidities, smoking status, number of medications, clinical frailty scale, deprivation index and number of contacts with telephone triage. Ethnicity was excluded from analysis due to the high proportion of missing data (22.2%). Obesity was excluded due to an observed implausible protective association with the primary outcome which we believe to be an artefact of how these data were collected and recorded in the electronic GP dataset. For those under 65 years, a frailty scale score of 1 was assigned, since the score is not validated in this age group.

To assess how accurately NHS 111 identified patients with adverse outcomes, the call disposition categories of the index contact were divided into a binary classification of either: ambulance dispatched, or other urgent clinical assessment required; and self-care or non-urgent assessment (online supplemental material 3). Urgent clinical assessment included advice to self-present to the ED, or provision of a further clinical assessment either immediately or within 4 hours of the call. Advice and call disposition provided by NHS 111 can change over successive calls as a patient’s condition changes. Therefore, to assess if deterioration was recognised over multiple calls, a sensitivity analysis was conducted in patients who had an adverse outcome in which the disposition of the call immediately before the adverse outcome was used for binary classification.

We assessed the accuracy of the binary triage classification (ambulance dispatch/urgent clinical assessment vs self-care/non-urgent assessment) in terms of sensitivity, specificity, positive predictive value (PPV) and negative predictive value (NPV) for the primary outcome with 95% CIs. To assess whether the implementation of different COVID-19-related NHS Pathways affected the accuracy of triage, accuracy was estimated for the whole study period and in two distinct time periods. The first time period (18 March 2020 to 2 June 2020) encompassed the use of Pathways 19.3.3/4/5/7 by YAS and the second period (2 June 2020 (10:30 hours) to 29 June 2020) Pathways 19.3.8/9 which incorporated loss of taste or smell as a feature of COVID-19 infection (online supplemental material 4).

Patient characteristics of false negatives (those advised to self-care/non-urgent assessment who experienced the primary outcome) and true positives (those provided with an ambulance/urgent assessment who experienced the primary outcome) were compared. Similarly, we compared the characteristics of false positives (those provided with an ambulance/urgent assessment and not conveyed to hospital and did not experience the primary outcome) and true negatives (those advised to self-care/non-urgent assessment) among those who did not experience the primary composite adverse outcome. In patients with the adverse outcome, multivariable logistic regression was used to identify patient characteristics associated with false negative triage. We completed equivalent analysis in those without the adverse outcome to identify factors which predicted false positive triage. The models included: age, gender, available comorbidities, smoking status, number of medications, deprivation index and number of contacts with telephone triage. Due to a low proportion of missing data in included variables, complete case analysis was conducted. As with the previous analysis, ethnicity and obesity were excluded. Frailty was additionally excluded from this modelling due to a high proportion of missing data (39.4% of false negatives).

The sample size was based on the number of NHS 111 calls for suspected COVID-19 that YAS received during the first wave of the pandemic. All multivariable logistic models included a sample size of >500 and >10 events (adverse clinical outcome, false positive or false negative triage) per predictor parameter.^22 23^ All totals presented are rounded to the nearest 5, with small numbers suppressed to comply with NHS Digital data disclosure guidance.

### Patient and public involvement

The Sheffield Emergency Care Forum (SECF) is a public representative group interested in emergency care research.^24^ Members of SECF advised on the development of the PRIEST study and two members joined the Study Steering Committee. A PRIEST study patient public involvement (PPI) group was created during the study which included patients who had been admitted to hospital with COVID-19 or their family members. Although not involved in conducting the analyses, both PPI groups were consulted regarding study design, particularly the ethical implications of using routine health data for research. All study findings were presented and discussed with the PPI groups. Members helped with interpretation of findings particularly regarding acceptable risk of misclassification.

## Results

### Study population

**Figure 1:**
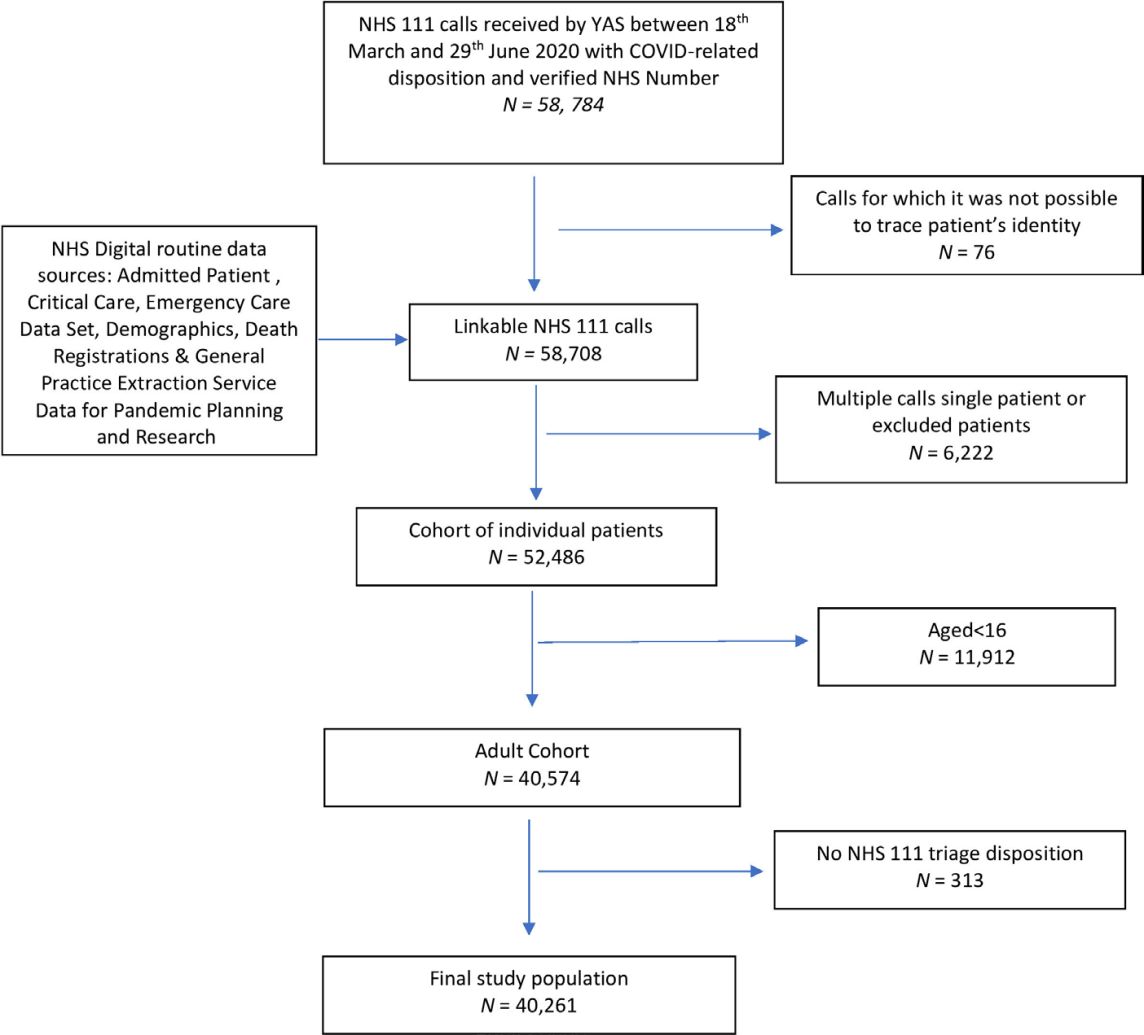
Strengthening the Reporting of Observational Studies in Epidemiology flow diagram of selection of study population. NHS, National Health Service; YAS, Yorkshire Ambulance Service.

Figure 1 and table 1 summarise study cohort derivation and the characteristics of the 40 261 included individuals. In total, 1200 people (3%, 95% CI: 2.8% to 3.2%) experienced the primary outcome (death or organ support) within 30 days following first contact with telephone triage services and 670 (56%) of adverse outcomes occurred within 7 days of contact. In our study cohort, 8165 patients (20.3%, 95% CI: 19.9% to 20.7%) were conveyed or self-presented to the ED and 4490 (11.2%, 95% CI: 10.9% to 11.5%) were admitted as hospital inpatients within 30 days of index contact.

**Table 1:** Population characteristics

Population characteristic	Level	Whole population n=40 261	Primary outcome n=1200
**Age (years**)	Median (IQR)* Mean	47 (32–61) 48.4	77 (63–86) 73.3
**Gender (n, %)**	Male	17 5645 (43.6%)	710 (59%)
**Comorbidity (n, %)**	Cardiovascular disease	850 (2.1%)	80 (6.6%)
	Chronic respiratory disease	10 315 (25.6%)	320 (26.7%)
	Diabetes	4245 (10.5%)	275 (22.8%)
	Hypertension	7275 (18.1%)	510 (42.6%)
	Immunosuppression (including steroid use)	3320 (8.2%)	350 (29.3%)
	Active malignancy	460 (1.2%)	55 (4.6%)
	Obesity	6205 (15.4%)	105 (8.8%)
	Pregnant	685 (1.7%)	‡
	Renal impairment	415 (1%)	45 (3.7%)
	Smoker	11 160 (29%)	1200 (35.9%)
	Stroke	230 (0.6%)	20 (1.8%)
**Number of drugs used** (**n, %)**	0	17 830 (44.3%)	230 (19.2%)
	1–5	17 300 (43%)	620 (51.5%)
	6–10	4550 (11.3%)	315 (26.5%)
	11 or more	585 (1.5%)	40 (3.3%)
**Clinical frailty scale** (**N, %)**	Unknown	6610 (16.4%)	605 (50.3%)
	Aged <65 years	31 860 (79.1%)	330 (27.4)
	1–3	200 (0.5%)	10 (0.9%)
	4–6	670 (1.7%)	60 (5.1%)
	7–9	925 (2.3%)	195 (16.3%)
**Ethnicity (n, %)**	Unknown	8950 (22.2%)	410 (34%)
	Asian or Asian British	4335 (10.8%)	90 (7.3%)
	Black or black British	850 (2.1%)	15 (1.3%)
	Mixed	540 (1.3%)	10 (0.8%)
	Other ethnic groups	615 (1.5%)	10 (1%)
	White	24 975 (62%)	670 (55.7%)
**Deprivation index (n, %)**	Unknown	2360 (5.9%)	135 (11.2%)
	1–2	14 355 (35.7%)	330 (27.6%)
	3–4	7365 (18.3%)	205 (17.1%)
	5–6	6005 (14.9%)	180 (15%)
	7–8	5835 (14.5%)	195 (16.1%)
	9–10	4345 (10.8%)	155 (13%)
**Index triage category (n, %)**	Ambulance dispatch/Urgent clinical assessment	15 030 (39.6%)	890 (74.2%)
	Ambulance response	3955 (9.8%)	450 (37.6%)
	Urgent follow-up GP assessment	555 (1.4%)	45 (0.4%)
	Urgent follow-up COVID-19 clinical assessment	11 235 (27.9%)	395 (32.7%)
	Self-care/Non-urgent assessment	24 335 (60.4%)	310 (25.8%)
	Self-care	12 925 (32.1%)	85 (7.2%)
	Non-urgent GP assessment	880 (2.2%)	10 (0.1%)
	Further non-urgent COVID-19 assessment	10 510 (26.1%)	215 (17.7%)
**Outcome (n, %)**	Death	910 (2.3%)	910 (75.6%)
	Deaths due to COVID-19 (including after 30 days)	710 (1.8%)	580 (48.3%)
	Organ support (within 30 days)	425 (1.1%)	425 (35.2%)
**Hospitalisation (n, %)**	ED attendance	8165 (20.3%)	840 (70%)
	Inpatient admission	4490 (11.2%)	840 (69.9%)
**Confirmed hospital diagnosis of COVID-19†**	In ED or as inpatient at 30 days	2370 (5.9%)	650 (54%)
**Number of NHS 111 contacts in study period (n, %)**	1	36 480 (90.6%)	1060 (88.2%)
	2	3035 (7.5%)	120 (9.7%)
	3 or more	750 (1.9%)	25 (2.1%)
**Time to primary outcome from index contact—up to and including (n, %)**	72 hours	320 (0.8%)	320 (26.5%)
	7 days	670 (1.7%)	670 (56%)

*IQR.

†Unrestricted community testing for suspected COVID-19 infection was only available from 18 May 2020. Confirmed diagnosis is based on inpatient PCR testing or clinical diagnosis in hospital.

‡Suppressed due to small numbers.

ED, emergency department; NHS, National Health Service.

The median age of the whole cohort was 47 years, the cohort had a higher proportion of females (56.4%) than males and had high rates of comorbidity (chronic respiratory disease 25.6%, diabetes 10.5% and hypertension 18.1%). In multivariable modelling (online supplemental material 5), known predictors of adverse outcomes including increasing age (1-year increase, OR 1.06, 95% CI: 1.06 to 1.07), male gender (female, OR 0.48, 95% CI: 0.40 to 0.58), diabetes (OR 1.62, 95% CI: 1.26 to 2.09) and frailty (moderate, OR 1.07, 95% CI: 0.71 to 1.07: severe, OR 2.51, 95% CI: 1.74 to 3.61) were associated with an increased risk of the primary composite adverse outcome.

### Accuracy of NHS 111 triage

A triage disposition of ambulance dispatch/urgent clinical assessment achieved a sensitivity of 74.2% (95% CI: 71.6% to 76.6%) to the primary outcome across the whole study period (table 2). If advised to self-care/non-urgent clinical assessment, the chance of experiencing an adverse outcome was approximately 1% (NPV: 98.7%, 95% CI: 98.6% to 98.9%). For patients who contacted NHS 111 multiple times, classification of the triage disposition on the basis of the last call before the primary outcome, instead of index contact, did not noticeably affect these estimates (sensitivity: 77.3%, 95% CI: 74.8% to 79.6% and (NPV: 98.9%, 95% CI: 98.7% to 99%).

**Table 2:** Performance of binary NHS 111 triage (ambulance or urgent assessment 4 hours or less) for composite outcome (death or organ support)

Adverse outcome up to 30 days (3%, 2.8%–3.2%) Whole study period			
N=40 261	**Adverse outcome**	**No adverse outcome**	
Ambulance/Urgent assessment	890	15 035	Sensitivity 74.2% (71.6%–76.6%) Positive predictive value 5.6% (5.2%–6%)
Self-care/Non-urgent assessment	310	24 025	Specificity 61.5% (61%–62%) Negative predictive value 98.7% (98.6%–98.9%)
**Adverse outcome up to 30 days (3.1%, 2.9%–3.2%**) **NHS triage pathways 19.3.3/4/5/7 (18 March 2020 to 2 June 2020**)			
N=36 124	**Adverse outcome**	**No adverse outcome**	
Ambulance/Urgent assessment	807	13 079	Sensitivity 73.2% (70.4%–75.7%) Positive predictive value 5.8% (5.4%–6.2%)
Self-care/Non-urgent assessment	296	21 942	Specificity 62.7% (62.1%–63.2%) Negative predictive value 98.7 (98.5%–98.8%)
**Adverse outcome up to 30 days (2.4%, 1.9%–2.9%**) **NHS triage pathways 19.3.8/9 (2 June 2020 to 30 June 2020**)			
N=4137	**Adverse outcome**	**No adverse outcome**	
Ambulance/Urgent assessment	84	1957	Sensitivity 85.7% (76.9%–91.7%) Positive predictive value 4.1% (3.3%–5.1%)
Self-care/Non-urgent assessment	14	2082	Specificity 51.5% (50%–53.1%) Negative predictive value 99.3 (98.9%–99.6%)

NHS, National Health Service.

Sensitivity of triage disposition was higher for adverse outcomes at 3 days from index contact (81.4%, 95% CI: 76.6% to 85.5%) (online supplemental material 6), than at 7 and 30 days. Specificity was comparable for adverse outcomes at 30 days (61.5%, 95% CI: 61% to 62%) and 3 days (60.8%, 95% CI: 60.2% to 61.3%). In the later period of NHS 111 clinical assessment pathway implementation, sensitivity to adverse outcomes at 30 days increased (85.7%, 95% CI: 76.9% to 91.7%) but this was associated with a reduction in specificity (51.5%, 95% CI: 50% to 53.1%) (table 2).

### Prediction of false negative or false positive triage

Online supplemental material 7 compares the characteristics of who were correctly triaged as true positives or misclassified as false negatives. In both groups, approximately 50% of people experienced the primary adverse outcome within 7 days of first contact, although a higher proportion of true positives experienced the adverse outcome within 3 days of contact. Multivariable modelling showed that younger age, multiple contacts and diabetes were associated with increased risk of false negative triage (table 3). The effect estimates for multiple NHS 111 contacts were similar if the triage disposition of last call before the primary outcome (two contacts, OR 1.96, 95% CI: 1.11 to 3.48 and three or more contacts, OR 7.78, 95% CI: 1.02 to 59.43) was used to classify true positives and false negatives.

**Table 3:** Multivariable model predicting false negatives

Population characteristic	Level	OR (95% CI) N=1065
**Age (years**)	1-year increase	0.99 (0.98 to 1.00)
**Gender**	Female	1.13 (0.84 to 1.52)
**Comorbidity**	Cardiovascular disease	0.48 (0.20 to 1.16)
	Chronic respiratory disease	1.00 (0.69 to 1.45)
	Diabetes	1.66 (1.13 to 2.45)
	Hypertension	0.99 (0.70 to 1.40)
	Immunosuppression (including steroid use)	0.62 (0.38 to 1.01)
	Active malignancy	0.42 (0.15 to 1.23)
	Obesity	Not included
	Renal impairment	0.86 (0.38 to 1.97)
	Smoker	0.81 (0.58 to 1.12)
	Stroke	1.99 (0.63 to 6.28)
**Number of drugs used**	0	Reference
	1–5	1.13 (0.74 to 1.74)
	6–10	0.60 (0.33 to 1.10)
	11 or more	0.38 (0.11 to 1.27)
**Deprivation index**	1–2	Reference
	3–4	1.27 (0.84 to 1.93)
	5–6	1.03 (0.66 to 1.61)
	7–8	1.26 (0.82 to 1.93)
	9–10	1.38 (0.88 to 2.15)
**Number of 111 contacts in study period**	1	Reference
	2	1.77 (1.14 to 2.75)
	3 or more	4.03 (1.68 to 9.65)

Online supplemental material 8 compares the characteristics of patients who received false positive or true negative triage classification; 24.9% of the cohort were false positives and table 4 presents the results of multivariable modelling to identify factors associated with being a false positive. Increased risk of being a false positive was associated with chronic renal impairment, immunosuppression and chronic respiratory disease (table 4). Other predictors included older age, smoking, increased medication use and female gender (table 4).

**Table 4:** Multivariable model predicting false positives

Population characteristic	Level	OR (95% CI) N=32 195
**Age (years**)	1-year increase	1.01 (1.01 to 1.01)
**Gender**	Female	1.05 (1.01 to 1.10
**Comorbidity**	Cardiovascular disease	1.04 (0.86 to 1.26)
	Chronic respiratory disease	1.31 (1.22 to 1.40)
	Diabetes	0.84 (0.77 to 0.93)
	Hypertension	0.97 (0.90 to 1.05)
	Immunosuppression (including steroid use)	1.49 (1.36 to 1.64)
	Active malignancy	1.25 (0.97 to 1.61)
	Renal impairment	1.52 (1.17 to 1.97)
	Smoker	1.10 (1.04 to 1.16)
	Stroke	1.23 (0.87 to 1.75)
**Number of drugs used**	0	Reference
	1–5	1.13 (1.06 to 1.21)
	6–10	1.60 (1.42 to 1.81)
	11 or more	2.36 (1.82 to 3.07)
**Deprivation index**	1–2	Reference
	3–4	0.95 (0.89 to 1.02)
	5–6	0.95 (0.89 to 1.03)
	7–8	0.99 (0.92 to 1.07)
	9–10	1.05 (0.97 to 1.14)
**Number of 111 contacts in study period**	1	Reference
	2	0.86 (0.78 to 0.95)
	3 or more	0.71 (0.58 to 0.88)

## Discussion

### Summary

Our study showed that, during the study period, telephone triage achieved a sensitivity of 74.2% (95% CI: 71.6% to 76.6%) and specificity of 61.5% (95% CI: 61% to 62%) for the primary outcome. Telephone triage recommended self-care or non-urgent assessment for the majority (60%), with a very low but non-negligible risk of adverse outcome (1.3%). Sensitivity of telephone triage was higher for outcomes at 3 and 7 days (online supplemental material 6) than 30 days, and sensitivity appeared to be increased at the expense of specificity in the later period of clinical assessment pathway implementation (table 2). Users of the service who were identified with possible COVID-19 infection had a low (3%) risk of adverse outcome.

To identify factors which may affect accuracy of triage, we used multivariable analysis to identify predictors of false negative and false positive triage. The findings need cautious interpretation, given the limited information available during telephone triage, but suggest that some comorbidities (such as chronic respiratory disease) may be overappreciated as predictors of adverse outcome, while the association of diabetes with adverse outcome may be under-recognised. Perhaps most striking, is that multiple contacts with NHS 111, in which possible COVID-19 infection was identifed, was associated with false negative assessment, suggesting that repeat contacts may require a more urgent response.

### Comparison with previous literature

The available evidence assessing the accuracy of telephone triage for serious clinical outcomes, particularly for patients with suspected COVID-19, is limited. Existing studies evaluating similar telephone triage ‘hotlines’ in the USA have described service use or acceptability.^6 7^ The sensitivity and specificity of telephone triage found in our study to the composite primary outcome is similar to that reported for clinical tools used to triage patient acuity in the ED, at a point on the receiver operating characteristic curve with an equivalent balance of sensitivity and specificity.^25^ Previous evaluations of telephone triage and other forms of telemedicine in emergency care or COVID-19 have largely assessed diagnostic accuracy of triage in identifying specific conditions.^26–29^ However, a systematic review of accuracy of emergency medical service dispatch by call handlers found the most urgent ambulance dispatch priorities to have sensitivities ranging between 78% and 95.6% for time critical conditions and specificities ranging between 15.4% and 83.8%. Despite the reported sensitivities being higher than achieved by telephone triage in our study, the associated negative predictive values ranged from 95.4% to 96.9%, similar to that estimated in our study.

### Strengths and limitations

Although telephone triage has been recommended and widely used during the pandemic in the UK and the USA to risk assess patients with suspected COVID-19 to limit potential spread of infection, this appears to be the first evaluation of accuracy.^6 30^ We have used a large cohort of patients identified from routinely collected telephone triage records and linked this to nationally collected, patient-level healthcare records to provide robust outcome data. We have assessed performance in a cohort of patients with suspected infection which, in the absence of accurate universally available rapid COVID-19 diagnostic tests, reflects the population which urgent and emergency care services must clinically triage. Unrestricted community testing for those with symptoms suggestive of COVID-19 infection was only available from 18 May 2020 and therefore it is not possible to estimate the proportion of confirmed infections. However, known factors associated with adverse outcomes in COVID-19 infection were found to be predictive of the primary outcome in our cohort including increasing age, male gender, diabetes and frailty.^31–33^

Due to the use of routinely collected data, there were high rates of missing data for some variables, for example, ethnicity and frailty, which prevented inclusion in some analyses. We have also assumed that if comorbidities were not recorded in the previous 12 months they were not present. The mechanism of how data are collected and recorded in the routine datasets used means that, as identified for obesity, there may be bias in the classification of patients. The estimated prevalence of obesity in our cohort is 15% (half that reported in the national health survey) and, as weight is not comprehensively and consistently measured by GPs, the observed protective association is likely to reflect unknown characteristics associated with a measurement being taken, rather than obesity itself.^34^

We have evaluated the performance of NHS 111 telephone triage as implemented by YAS. Although NHS 111 Pathways software algorithms are developed nationally, there may be variability in local implementation which may affect accuracy. During the study period, calls were diverted between regions and to a national centre due to excess demand. The basis on which calls were selected for diversion is not transparent, but it is possible that patients with less complex healthcare needs were diverted to the national centre, potentially affecting the generalisability of our results. Our study period includes multiple pathway iterations but, due to how rapidly assessment pathways were updated, it was not possible to assess the accuracy of individual assessment pathways (online supplemental material 4). A national online assessment tool was implemented from the end of February 2020 and this may have affected the characteristics of the population using telephone triage services for advice.^35^ However, it was not until June 2020 that the public were advised to use the NHS 111 online coronavirus service before calling NHS 111.

### Implications

Telephone triage performed comparably to triage methods used for patient acuity in the ED and, given the limited information available, including a lack of physiological parameters, this may reflect the best accuracy that could be achieved.^25 36^ It is difficult to accurately model the impact on emergency medical services if telephone triage had not been recommended for the initial assessment of patients with suspected COVID-19. However, in 2019, the estimated population of Yorkshire and the Humber was 5 502 967 (including children).^37^ On the basis of the number of patients in our cohort and study period, not using telephone triage could have led to around 61 extra ambulances or urgent clinical assessments being provided each day per 1 000 000 population, without considering diversion to the national centre. YAS provided a face-to-face response to an estimated 298 incidents per day in March 2020.^38^ NHS 111 telephone triage appears to have effectively helped to mitigate the risk of emergency healthcare services being overwhelmed by lower risk patients during the ‘first wave’ of the pandemic in England.

This must be weighed against the small but non-negligible risk that patients who were recommended to self-care or have a non-urgent clinical assessment had of serious adverse outcomes. Early clinical guidelines for the risk stratification of patients with suspected COVID-19 infection, on the basis of previous influenza epidemics, emphasised the importance of respiratory comorbidities and may have underestimated the risk associated with gender and diabetes.^39^ The results of our multivariable modelling reflects this, with the importance of smoking and chronic respiratory disease appearing to be overestimated and diabetes underestimated. Later clinical guidelines incorporated this evolving research base and emphasised the risk associated with diabetes.^40^ However, the association we found with multiple NHS 111 COVID-19-related contacts and risk of undertriage does not appear to have been previously identified and may reflect that patients with repeat contacts represent an unrecognised high-risk group. Patients with early representation after discharge from the ED are considered clinically high risk for adverse outcomes and misdiagnosis and this is likely to be reflected in patients who contact NHS 111.^41^ This finding has been fed back to the telephone triage service provided by YAS and is likely to be applicable to telephone triage in different settings.

Telephone triage services for suspected COVID-19 and other conditions have rapidly expanded during the pandemic across different settings, with specific COVID-19 telephone triage ‘hotlines’ created in parts of the USA.^6 7 42^ Different models for telephone triage in urgent and emergency care exist internationally.^26 43 44^ Research is needed to determine the optimal configuration of such services in terms of accuracy and cost-effectiveness.^43^ NHS 111’s use of trained, non-clinical call advisors for initial assessment contrasts with other national triage services, where assessments are performed by nurses and other clinicians: this may impact accuracy, acceptability and cost.^44^ The acceptable risk of deterioration following such triage is subjective and significant variation in risk tolerance between clinicians and public representatives has been demonstrated.^45^ Research may be needed to support implementation of telephone triage methods and tailor triage to the resource constraints and risk tolerance of different healthcare settings. Within the context of the UK, future research could use our methods for a national evaluations of NHS 111 performance, including the devolved nations, and to assess regional variations in triage, accuracy and safety.

## Conclusions

We have conducted the first evaluation of accuracy of telephone triage for need for emergency treatment in patients with suspected COVID-19 infection. Telephone triage appears to have had an important role in managing lower-risk patients and potentially preventing many patients who required no specific treatment from attending hospitals or other care providers. This must be weighed against the small but non-negligible risk of serious adverse outcomes in patients advised to self-care or have a non-urgent clinical assessment. Repeat contact with triage services may need more recognition as an important predictor of subsequent deterioration. Future research is needed to determine acceptable risk of deterioration in patients advised to self-care and the optimal configuration of telephone triage services.

## Data Availability

Data may be obtained from a third party and are not publicly available. The data used for this study are subject to data sharing agreements with NHS Digital and Yorkshire Ambulance Service, which prohibits further sharing of individual level data. The datasets used are obtainable from these organisations subject to necessary authorisations and approvals.
