# Supplementary material for: Accuracy of telephone triage for predicting adverse outcomes in suspected COVID-19: an observational cohort study
Source: BMJ Qual Saf. 2022 Mar 30;33(6):375–85. doi: 10.1136/bmjqs-2021-014382 (PMC8983415; doi:10.1136/bmjqs-2021-014382)
Supplement: Supplementary data [file bmjqs-2021-014382supp001.pdf]

Supplementary Material 1: Variables provided by YAS based on electronic call records

Variable
NHS Number
Age
Gender
Call date and time (used to order multiple calls by individual)
NHS 111 pathway used
Disposition (Outcome) of Call

Supplementary Material 2: Variables provided by NHS digital

Variable	Source	Timeframe
Date of Birth (single source of truth to calculate age)	Any NHS digital dataset	
Gender	Any NHS digital dataset	
Post code (used to calculate deprivation index)	Any NHS digital dataset	
Ethnicity	Any NHS digital dataset	
Date of death	Office for National Statistics Mortality Data	Following first contact NHS 111 in study period
Cause of death (ICD10 code)	Office for National Statistics Mortality Data	Following first contact NHS 111 in study period
Date of critical care admission	Hospital Episode Statistics Critical Care Inpatient Data	Following first contact NHS 111 in study period
Basic or advanced renal, cardiovascular or respiratory support during critical care admission	Hospital Episode Statistics Critical Care Inpatient Data	Following first contact NHS 111 in study period
Date of Inpatient admission	Hospital Episode Statistics Admitted Patient Care record	Following first contact NHS 111 in study period
Date of COVID-19 diagnosis (Inpatient)	Hospital Episode Statistics Admitted Patient Care record	Following first contact NHS 111 in study period
Date of Emergency Department Attendance	Hospital Episode Statistics Emergency Care record	Following first contact NHS 111 in study period
Date of COVID-19 diagnosis (Emergency Department)	Hospital Episode Statistics Emergency Care record	Following first contact NHS 111 in study period
Cardiovascular Comorbidity	GPES Data for Pandemic Planning and Research	1 year before first contact NHS 111 in study period
Respiratory Comorbidity	GPES Data for Pandemic Planning and Research	1 year before first contact NHS 111 in study period
Diabetic Comorbidity	GPES Data for Pandemic Planning and Research	1 year before first contact NHS 111 in study period
GP estimated Frailty	GPES Data for Pandemic Planning and Research	1 year before first contact NHS 111 in study period
Hypertension	GPES Data for Pandemic Planning and Research	1 year before first contact NHS 111 in study period

Immunosuppression	GPES Data for Pandemic Planning and Research	30 days before first contact NHS 111 in study period
Malignancy	GPES Data for Pandemic Planning and Research	1 year before first contact NHS 111 in study period
Obesity	GPES Data for Pandemic Planning and Research	1 year before first contact NHS 111 in study period
Pregnancy	GPES Data for Pandemic Planning and Research	9 months before first contact NHS 111 in study period
Renal Impairment	GPES Data for Pandemic Planning and Research	1 year before first contact NHS 111 in study period
Smoking Status	GPES Data for Pandemic Planning and Research	Last recorded
Drug Count	GPES Data for Pandemic Planning and Research	Last recorded

Supplementary Material 3: Classification 111 triage categories

Ambulance dispatched or other urgent clinical assessment	Self-care or non-urgent assessment
Ambulance response<br>Speak to a Clinician from our service Immediately<br>COVID risk Clinical Assessment service 1 hour<br>COVID risk Clinical Assessment service 2 hours<br>COVID risk Clinical Assessment service 4 hours<br>Speak to a Primary Care Service within 1 Hour<br>Speak to a Primary Care Service within 2 Hours<br>Advised to make own way for urgent clinical assessment	COVID Self Care<br>COVID Coordination Service<br>COVID risk Clinical Assessment service 6 hours<br>COVID risk Clinical Assessment service 12 hours<br>COVID risk Clinical Assessment Service next working day<br>Home Management<br>The call is closed with no further action needed<br>All Dental Dispositions<br>Any disposition to contact own GP or primary care service<br>Midwife assessment

Supplementary Material 4: NHS 111 COVID-19 assessment pathways implementation by Yorkshire Ambulance Service

NHS 111 Pathway*	Implementation Date	Study Period	Description
19.3.3	16/3/2020	1	First specific COVID-19 pathway, focus on remote consultations or remote follow-up.
19.3.4	Not implemented	1	
19.3.5 & 19.3.6	03/04/2020	1	Chest pain incorporated as part of COVID assessment pathway<br>New questions to identify vulnerable Patients
19.3.7	10/04/2020	1	New advice provided for signs of deterioration and what to do in patients advised to self-care.
19.3.8	02/06/2020	2	More specific triage for symptoms non-specific for COVID e.g. cough or fever offers normal triage plus access to coronavirus triage if certain trigger criteria were met.
19.3.9	03/06/2020	2	Incorporates loss of taste or smell as COVID symptom

*Information regarding specific pathways obtained here https://digital.nhs.uk/services/nhs-pathways/nhs-pathways-service-information/clinical-release-notes/archived-clinical-release-notes/2020-archived-clinical-release-notes

Supplementary Material 5: Multi-variable model predicting primary outcome

Population Characteristic	Level	Odds ratio (95% Confidence Interval) N= 31, 820
**Age (Years)**	1-year increase	1.06 (1.06 to 1.07)
**Gender (N, %)**	Female	0.48 (0.40 to 0.58)
**Comorbidity (N, %)**	Cardiovascular Disease	0.80 (0.51 to 1.26)
	Chronic Resp. Disease	0.96 (0.76 to 1.21)
	Diabetes	1.62 (1.26 to 2.09)
	Hypertension	1.08 (0.85 to 1.38)
	Immunosuppression (including steroid use)	0.97 (0.74 to 1.28)
	Active Malignancy	1.32 (0.80 to 2.19)
	Obesity	Not included
	Renal Impairment	1.21 (0.69 to 2.13)
	Smoker	0.85 (0.69 to 1.04)
	Stroke	0.54 (0.20 to 1.41)
**Number of Drugs Used (N, %)**	0	Reference
	1-5	1.03 (0.80 to 1.36)
	6-10	0.93 (0.63 to 1.39)
	11 or more	0.87 (0.46 to 1.64)
**Clinical Frailty Scale (N, %)**	1-3	Reference
	4-6	1.07 (0.71 to 1.61)
	7-9	2.51 (1.74 to 3.61)
**Deprivation Index (N, %)**	1-2	Reference
	3-4	1.04 (0.80 to 1.34)
	5-6	1.02 (0.77 to 1.34)
	7-8	1.11 (0.85 to 1.45)
	9-10	1.09 (0.81 to 1.46)
**Number of 111 contacts in study period (N, %)**	1	Reference
	2	1.69 (1.27 to 2.27)
	3 or more	2.73 (1.70 to 4.39)

Supplementary Material 6: Performance of binary NHS 111 triage (ambulance or urgent assessment 4 hours or less) for composite outcome (death or organ support)

**CompositeAdverse outcome 7 days (1.7%, 1.6-1.8%)**			
N=40, 261	**Adverse Outcome**	**No Adverse Outcome**	
Ambulance/urgent assessment	500	15,430	Sensitivity 74.4% (70.9-77.6%)<br>Positive Predictive Value 3.1% (2.9 – 3.4%)
Self-care/ non-urgent assessment	170	24,160	Specificity 61% (60.5% - 61.5%)<br>Negative Predictive Value 99.3% (99.2 - 99.4%)

**Composite Adverse outcome 72 hours (0.8%, 0.7-0.9%)**			
N=40, 261	**Adverse Outcome**	**No Adverse Outcome**	
Ambulance/urgent assessment	260	15,670	Sensitivity 81.4% (76.6-85.5%)<br>Positive Predictive Value 1.6% (1.4 – 1.8%)
Self-care/ non-urgent assessment	60	24,275	Specificity 60.8% (60.3% - 61.3%)<br>Negative Predictive Value 99.8% (99.7 - 99.9%)

Supplementary 7: Comparison of False Negatives and True positives

Population Characteristic	Level	False Negatives (30 days) N= 310	True Positives (30 days) N=890
**Age (Years)**	Median (IQR)*<br>Mean	71.5 (57-84)<br>69.9	78 (66-86)<br>74.5
**Gender (N, %)**	Male	185<br>(59%)	525<br>(58.9%)
**Comorbidity (N, %)**	Cardiovascular Disease	**	70<br>(8%)
	Chronic Respiratory Disease	70<br>(22.9%)	250<br>(28.1%)
	Diabetes	80<br>(24.8%)	200<br>(22.1%)
	Hypertension	115<br>(36.7%)	400<br>(44.7%)
	Immunosuppression	30<br>(8.7%)	160<br>(18.1%)
	Active Malignancy	**	50<br>(5.5%)
	Obesity	40<br>(12.3%)	70<br>(7.6%)
	Pregnant	**	**
	Renal Impairment	**	35<br>(4%)
	Smoker	95<br>(31%)	335<br>(37.6%)
	Stroke	**	15<br>1.7% (1-2.8%)
**Number of Drugs Used (N, %)**	0	75 (23.6%)	160 (17.6%)
	1-5	175 (56.5%)	445 (49.7%)
	6-10	55 (18.1%)	260 (29%)
	11 or more	5 (1.9%)	33 (3.7%)
**Clinical Frailty Scale (N, %)**	Unknown	130 (42.6%)	470 (53%)
	Aged<65	120 (39.4%)	210 (23.2%)
	1-3	**	10 (1%)
	4-6	**	45 (4.9%)
	7-9	40 (11.9%)	160 (17.9%)
**Ethnicity (N, %)**	Unknown	100 (31.3%)	310 (34.9%)
	Asian or Asian British	30 (10%)	10 (1%)
	Black or Black British	**	**
	Mixed	**	10 (1%)
	Other Ethnic Groups	**	60 (6.4%)
	White	170 (55.2%)	500 (55.9%)
**Deprivation Index (N, %)**	Unknown	30 (10%)	105 (11.7%)
	1-2	85 (27%)	250 (28%)
	3-4	55 (18.1%)	150 (16.7%)

	5-6	40 (13.6%)	140 (15.5%)
	7-8	50 (16.8%)	140 (15.8%)
	9-10	45 (14.8%)	110 (12.4%)
**Index Triage Category (N, %)**	Self-care	85 (27.7%)	NA
	Ambulance Response	NA	450 (50.6%)
	Further COVID Assessment	215 (68.7%)	295 (44.1%)
	Further GP Assessment	10 (3.2%)	45 (5.1%)
**Outcome (N, %)**	Death	210 (67.1%)	700 (78.6%)
	Deaths due to COVID (including after 30 days)	140 (45.8%)	435 (48.8%)
	Organ support (within 30 days)	140 (45.2%)	225 (45.2%)
**Hospitalisation (N, %)**	Emergency Department (ED) Attendance	210 (68.4%)	630 (70.6%)
	Inpatient admission	215 (69%)	625 (70.3%)
**Diagnosis of COVID**	In ED or as inpatient at 30 days	170 (55.5%)	475 (53.4%)
**Number of NHS 111 contacts in study period (N, %)**	1	250 (81.3%)	810 (90.6%)
	2	44 (14.2%)	75 (8.2%)
	3 or more	15 (5%)	10 (1.2%)
**Time to Primary Outcome (N, %)**	72 hours	60 (19%)	260 (29.1%)
	7 days	170 (55.5%)	500 (56.1%)

*Interquartile Range (IQR)

**Numbers suppressed due to small numbers

Supplementary Material 8: Comparison of False Positives and True Negatives

Population Characteristic	Level	False Positives (30 days) N= 10, 000	True Negative (30 days) N=24, 025
**Age (Years)**	Median (IQR)*<br>Mean	49 (33-65)<br>49.9	44 (31-57)<br>45.2
**Gender (N, %)**	Male	4,190<br>(41.9%)	10,460<br>(43.5%)
**Comorbidity (N, %)**	Cardiovascular Disease	235<br>(2.4%)	310<br>(1.3%)
	Chronic Respiratory Disease	3,160<br>(31.6%)	5,125<br>(21.3%)
	Diabetes	1,120<br>(11.2%)	2,100<br>(8.7%)
	Hypertension	2,010<br>(20.1%)	3,320<br>(13.8%)
	Immunosuppression	1070<br>(10.7%)	1, 278<br>(5.3%)
	Active Malignancy	120<br>(1.2%)	150<br>(0.6%)
	Obesity	1,660<br>(16.6%)	3,490<br>(14.5%)
	Pregnant	190<br>(1.9%)	470<br>(2%)
	Renal Impairment	135<br>(1.4%)	140<br>(0.6%)
	Smoker	3, 245<br>(32.4%)	6, 200<br>(25.8%)
	Stroke	70<br>(0.7%)	85<br>(0.35%)
**Number of Drugs Used (N, %)**	0	3, 900 (39%)	12, 230 (50.9%)
	1-5	4, 500 (45%)	9, 830 (40.9%)
	6-10	1, 420 (14.2%)	1, 800 (7.5%)
	11 or more	190 (1.9%)	165 (0.7%)
**Clinical Frailty Scale (N, %)**	Unknown	1,965 (19.6%)	2,790 (11.6%)
	Aged<65	7,470 (74.7%)	20,725 (86.3%)
	1-3	60 (0.6%)	85 (0.4%)
	4-6	225 (2.2%)	220 (0.9%)
	7-9	290 (2.9%)	210 (0.9%)
**Ethnicity (N, %)**	Unknown	2,100 (21%)	5,480 (22.8%)
	Asian or Asian British	1,025 (10.3%)	2,790 (11.6%)
	Black or Black British	190 (1.9%)	555 (2.3%)
	Mixed	110 (1%)	355 (1.5%)
	Other Ethnic Groups	130 (1.3%)	420 (1.7%)
	White	6,445 (64%)	14,420 (60%)
**Deprivation Index (N, %)**	Unknown	650 (6.5%)	1, 180 (4.9%)
	1-2	3, 570 (38.2%)	8, 730 (38.2%)
	3-4	1, 770 (19%)	4, 500 (19.7%)
	5-6	1, 470 (15.7%)	3, 665 (16%)
	7-8	1, 435 (15.4%)	3, 440 (15.1%)
	9-10	1, 105 (11.8%)	2, 510 (11%)

**Index Triage Category (N, %)**	Self-care	NA	12, 840 (53.4%)
	Ambulance Response	1, 645 (16.5%)	NA
	Further COVID Assessment	7, 935 (79.3%)	10, 300 (42.9%)
	Further GP Assessment	360 (3.6%)	490 (2%)
**Number of NHS 111 contacts in study period (N, %)**	1	9,230 (92.3%)	21,855 (91%)
	2	645 (6.5%)	1,740 (7.3%)
	3 or more	130 (1.3%)	430 (1.8%)

*Interquartile Range (IQR)
